# Supplementary material for: An after-action review of COVID-19 cases and mitigation measures at US Mission India, March 2020-July 2021
Source: PLOS Glob Public Health. 2025 May 20;5(5):e0003982. doi: 10.1371/journal.pgph.0003982 (PMC12091728; doi:10.1371/journal.pgph.0003982)
Supplement: Attachment — Submitted filename: Rebuttal Letter.docx [file pgph.0003982.s001.docx]

**Rebuttal Letter**

Dear Editor,

Thank you for the opportunity to revise our manuscript titled “An After-Action Review of COVID-19 Cases and Mitigation Measures at US Mission India, March 2020-July 2021” (New Manuscript ID: PGPH-D-24-02462, previous manuscript ID: PGPH-D-23-01590). We appreciate the constructive feedback on our previous submission from your editors and reviewers. We have carefully considered their comments and suggestions, in the revised manuscript and we are grateful for the chance to improve our work. Below, we outline our responses to major comments in the previous submission.

**1. Observation taken from the body of the email** - While the manuscript is interesting as it provides an overview of some of the mitigation measures implemented and cases of COVID-19, the conclusions are not in fact supported by the data presented (that the mitigation strategies were well planned and that they prevented transmission of COVID-19). If this in fact the objective of the manuscript, a much more in-depth description of the measures are needed and consider using the TIDier guidelines for intervention description and an impact evaluation (effectiveness) of the measures should be done using quasi-experimental methods (use of external control group).

**Response:** We acknowledge and appreciate this comment. Our manuscript is an after-action review and description of COVID-19 cases in US Mission India and associated mitigation activities. We describe a real time response in one of the largest US Missions in the world. This was not an interventional study or analytical study as mentioned in the abstract and introduction sections of the manuscript therefore data was reviewed retrospectively. The revised manuscript and abstract highlights the conclusion more clearly.

**Major Observations:**

**2.** It appears that there's no reason for this review, or if any at all, it needs to be clearly defined towards the last paragraph of the introduction.

**Response:** We acknowledge and appreciate this comment. US Mission India is among the largest in the world. During the early months of the COVID-19 response, the Mission worked to evacuate many Americans and the remaining Mission staff worked to protect the health of the Mission community. This paper is an after-action review describing COVID-19 cases in US Mission India and the mitigation strategies across a unique population working for Mission India. We provide lessons learnt in standing up response structures during COVID-19 pandemic, which may be helpful in responding to similar pandemics (described in the introduction in lines 57-64).

**3.** The authors have not clearly identified/presented what is unique about this review's findings and the added value to future pandemic mitigation measures the scientific community.

**Response:**

Currently, there are around 70,000 people working across US Missions overseas. We describe the impact, mitigation strategies and outcomes of the COVID-19 pandemic in one of the largest Missions in the world. We believe our experience will be relevant to other large Missions in standing up a similar response. We describe the specific focus for this review in lines 57-64, 393-395, and 404-413.

**4.** Pages 33-36: Contain the Figures. So, it is unclear why the authors repeated the Figures in the main text. It is tantamount to duplication.

**Response:**

We acknowledge and appreciate this comment. We have removed some duplication and summarized all the able findings in the revised manuscript.

**5.** Lines 83-87: The nature of the review that was done is unclear- what were the review criteria? What exactly did you seek to find in these meetings, trainings, webinars, etc., and what variables were outlined to ensure that this was done accordingly? How were these variables measured? What were (if any) the primary and secondary outcomes and measures. Any covariates?

**Response:**

We acknowledge and appreciate this comment. Our manuscript is an after-action review and description of COVID-19 cases in US Mission India and associated mitigation activities. We describe a real time response in one of the largest US Missions in the world. We describe mitigation activities during the course of the pandemic, which including coordination meetings, trainings and webinars to help the community in India manage the pandemic. The revised manuscript mentions the methodology more clearly.

**6.** Lines 91-103: It is great that the authors provided the inclusion and exclusion criteria. However, it would be interesting to know how the authors ensured that some of those who tested positive within the exclusion criteria only contracted the virus from outside India, and those included only contracted the virus from within India. Were there instances that some of the cases included had epidemiological linkage to cases excluded or the vice versa, and how did they handle such cases? This has the potential to influence the final numbers included in the analysis and the findings thereof. Lines 118-120: What categorical variables were included here and why were these selected? Which were the covariates?

**Response:**

We acknowledge and appreciate this comment. In lines 90-93 we state that we tried to exclude patients who tested positive outside India while on a travel (anyone who is not physically in Indian territory was excluded).

Furthermore, if anyone tested positive or clinical diagnosis of COVID-19 was made while outside India, they were made to isolate as per country specific guidelines, so, they couldn’t travel back to India, so we would not have been able to include them.

**7.** Ethical considerations Lines 127-129: The authors need to provide a copy of the letter from the Office of the Associate Director for Science of US CDC, as evidence that this study did not require full institutional review. What was/were the outcome variable(s)?

**Response:**

We acknowledge and appreciate this comment. We have shared the letter with journal team in the beginning before the peer review process. We are sharing the letter again with the revised manuscript submission.

**8.** Results Lines 133-137: The authors briefly described the sociodemographic characteristics of their study participants but did not provide a frequency table to show the details. Instead, they presented Figure 1, which has not relation at all with the information provided in lines 133-137. It all looks very confusing.

**Response:**

We acknowledge and appreciate this comment and have incorporated the changes requested in the revised manuscript.

**9.** Were these cases influenced by the variant of concern of the season - how?

**Response:**

We acknowledge and appreciate this comment. Unfortunately, genotyping was not widely available at the early stages of the pandemic. The Government of India did provide an update on the dominant variant when genotyping became more wide spread, but we could not link cases in real time to specific variants.

**10.** How many of these cases were vaccinated (at least a dose of any approved COVID vaccine) or not? What was the overall vaccination coverage per season of dominant variant of concern - e.g., overall vaccination coverage in Delta was (CI:...)?

**Response:**

We acknowledge and appreciate this comment. Please note that our data collection was not targeted towards publication and was targeted towards organizational effort to combat COVID-19 transmission. The population we were dealing with was a dynamic population having access to vaccination from the US vaccination centers (while on a travel to the US), vaccination being given at the US Embassy and Government of India/ Private Hospital administering vaccinations, all running their own software for documenting doses administration, hence it was difficult to track the exact timing of vaccination and the booster doses in relation the COVID-19 illness and more so after the fact and it wasn’t part of the scope of our study. We have mentioned all the available vaccine related information in lines 164-168 and 357-358 and also the mentioned the limitations in line 399-402 in the revised manuscript.

**11.** Please provide a flow diagram of participants selection, beginning from Index cases notified during study period, cases included, then cases per Mission, etc., up to the final included for each.

**Response:**

We acknowledge and appreciate this comment. Within the confines of a retrospective review of data, we are able to describe the time distribution in each of US Embassy and Consulate as seen in figure 2.

**12.** Line 242 - Table 1: Please present information to include confidence intervals of the AR, and clinically significant difference. Were there any missing data?

**Response:**

We acknowledge and appreciate this comment. This has been complied in the revised manuscript Table 1.

**13.** Also provide a general Table showing the Characteristics of participants in a study of SARS-CoV-2 secondary attack rates Across US Mission India in grouped by year. You may (if feasible) include information on vaccinated and unvaccinated Mission contacts according to the prevailing dominant variant of the season.

**Response:**

We acknowledge and appreciate this comment. We describe patient characteristics in Table 2, Figure 3, Table 3, Table 4, Figure 4.

**14.** Line 248: The authors said, "...about 22% (138/625) of cases were asymptomatic." This is consistent with earlier presentation about the proportion of asymptomatic cases. However, the went ahead to say that, "Among the 498 symptomatic cases,...". This is very contradictory - where from the 498 asymptomatic cases suddenly?

**Response:**

We acknowledge and appreciate this comment. We have corrected this error. It should be 138/636, which is about 22%.

**15.** Line 252 - Table 2: Please include the CIs

**Response:**

We acknowledge and appreciate this comment. Complied in the edited manuscript Table 1.

**16.** Line 275 - Table 3: Please organize the table for clarity. The authors presented earlier that 57% of 167 cases with comorbidities had hypertension, and in Table 3 they presented comorbidities out of the overall of 627 cases instead of 167. So, the Table suggests that the 57% of hypertension constituted is denominated from the 627. This has the potential to easily mislead the reader as there is no clarity.

**Response:**

**Response:**

We acknowledge and appreciate this comment. So, 167/627 had co-morbidities, out of which 96 had hypertension which is 57% (96/167). The same has been described in table 3 and line 279.

**17.** Interestingly, the sum of the numbers for the tabulated comorbidities is 267, which is 100-points greater than the 167 the authors mentioned. Likewise, the sum of the corresponding percentages is more than 100%. These inconsistencies need to be addressed. If for any reason these inconsistencies are to be maintained as they are, it will benefit the reader if clarifications are added as footnotes.

**Response:**

We acknowledge and appreciate this comment. The variables in the table are not mutually exclusive. So, an important point to note is that one person can have more than one condition including co-morbidities (for example a person suffering from hypertension can have diabetes too). We have provided a foot note for better clarity in the revised manuscript.

**18.** Lines 340-341 - Table 4: The number of overall cases has been inconsistent throughout the manuscript. It is 636 in Table 1, 629 in Table 2, 627 in lines 262-266 and in Figure 5, and 634 in Figure 2. In Table 4, n=627 in the title, but the total positive cases across all consulates and across Office, Household, Social, and Family within the table is 624. Interestingly,

there has been no mention of missing data. At each point that the total positive cases are different from previously stated, the authors need to provide an explanation.

**Response:**

We acknowledge and appreciate this comment. In the line 399, we have mentioned about missing data in limitation section.

**19.** Lines 358-359: "One-third of cases had comorbidities." This statement seems counterproductive since one-third of 636 cases is 212. Thus, it contradicts the statement in line 263 where the authors mentioned that '"27% (167/627) had comorbidities"'. Please clarify.

**Response:**

We acknowledge and appreciate this comment. We corrected this now to read - “One-third of symptomatic cases had comorbidities” which is 33.5 % (167/498)

We have also made additional improvements to the manuscript based on the overall feedback and all minor comments from the reviewers, including making the manuscript crisper, adding clarifications to improve the understanding.

Thank you once again for your valuable comments. We believe that the revisions have strengthened our manuscript, and we look forward to your favorable consideration for publication.

Sincerely,

Dr Jaspreet Singh

US Embassy, New Delhi

Email – [dr.singh.jaspreet@outlook.com](mailto:dr.singh.jaspreet@outlook.com)

Phone - +91-99999-36899
