# Supplementary material for: An after-action review of COVID-19 cases and mitigation measures at US Mission India, March 2020-July 2021
Source: PLOS Glob Public Health. 2025 May 20;5(5):e0003982. doi: 10.1371/journal.pgph.0003982 (PMC12091728; doi:10.1371/journal.pgph.0003982)
Supplement: Attachment — Submitted filename: Rebuttal_Letter_auresp_1.docx [file pgph.0003982.s002.docx]

**Rebuttal Letter**

Dear Editor,

We thank you for the opportunity to revise our manuscript entitled “An After-Action Review of COVID-19 Cases and Mitigation Measures at US Mission India, March 2020-July 2021” (New Manuscript ID: PGPH-D-24-02462, previous manuscript ID: PGPH-D-23-01590). We acknowledge and appreciate your comments and feedback. Below, we outline our responses to all of the comments.

1. Authors need to correct grammatical errors in the manuscript. For example, in the abstract, there is a sentence, “We described the COVID-19 response across the US Mission India (US Embassy New Delhi, US Consulates – Mumbai, Hyderabad, Chennai and Kolkata) in order use the learnings for a possible future pandemic”. The authors can change it to “to use the learnings”

**Response:**

We acknowledge and appreciate this comment and have incorporated the changes. In addition, we reviewed the manuscript again for a thorough grammatical error check.

2. Abstract:
   Method – instead of “conducted a secondary data analysis”, the authors can consider writing “we analyzed the investigation and contact tracing program data”.

**Response:**

We acknowledge and appreciate this comment and have incorporated the changes.

3. The methods section in the abstract needs more information on data acquisition and analysis reflecting the analysis methods for the results given.

**Response:**

We acknowledge and appreciate this comment and have incorporated the changes in the abstract method section.

4. It is not clear what is meant by “Health unit” – The authors should consider making it clear in the abstract.

**Response:**

We acknowledge and appreciate this comment. We have defined Health Unit in the method section. Health Unit is an outpatient clinic at US Embassy and US Consulates in India.

5. Give the numerator for case fatality rate.

**Response:**

We acknowledge and appreciate this comment. We have added the numerator along with the case fertility rate as per the suggestion.

6. Does “25% positivity rate” among contacts refer to secondary attack rate? If yes, the authors can use the same term. Despite whether it is SAR or not, the authors need to explain what is this in the methods section.

**Response:**

We acknowledge and appreciate this comment. We revised it as contact positivity rate. We did this to avoid confusion between attack rate which we have put in the manuscript for various locations. The contact positivity rate is the secondary attack rate.

7. Results part is so big with so much data which does not link with the conclusion directly. For example, % of asymptomatic case-patients, number of clusters and the median time between symptoms and notification. The authors can consider giving more information in the methods instead of this information in the results.

**Response:**

We acknowledge and appreciate this comment. We have incorporated the more information in the method sections in the manuscript.

8. The authors need to make sure that methods, results and conclusions align with one another.

**Response:**

We acknowledge and appreciate this comment. We have incorporated the requested changes for alignment in the method and result sections.

9. Keywords to be arranged alphabetically and better to use MeSH words.
   **Response:**

We acknowledge and appreciate this comment. We have incorporated the requested changes.

10. Introduction:
    Make sure the objective is given in the past tense
    **Response:**

We acknowledge and appreciate this comment. We have incorporated the requested changes.

11. Methods:
    Explain the study site in detail. Give more details which are relevant to understand the interventions and data in the results – what is health unit? What is the administrative structure.

**Response:**

We acknowledge and appreciate this comment. We have defined Health Unit in the methods section.

12. Give the definition for cases and contacts.
    **Response:**

We acknowledge and appreciate this comment. We have incorporated the requested changes in the lines 90-107 under the section “Analysis of health unit case and contact investigation program”

13. What tests were used for COVID-19 testing? This should be part of the methods section.
    **Response:**

We acknowledge and appreciate this comment. Since this was a secondary data analysis, the testing was not a part our study. We have defined the case definition in the method section (lines 90-107). We have described the same in the discussion section.

14. Who maintained the data on surveillance and contact tracing within each US consulate and how was it consolidated?
    **Response:**

We acknowledge and appreciate this comment. We have incorporated the information in lines 87-89.

15. In data analysis – how giving the “frequency of case identification by Mission” can be an epidemic curve? It is not a time distribution.
    **Response:**

We acknowledge and appreciate this comment. The chart shows the distribution of cases by time in the US Mission India. We have removed the word “epidemic curve”.

16. Mention what is the source of data for cases in India, Delhi, Chennai, Hyderabad, Kolkata and Mumbai.
    **Response:**

We acknowledge and appreciate this comment. We have incorporated the data source in the method section (line 107-109).

17. Results:
    The control activities are mentioned in detail. However, it is not clear who was responsible for each activity. For example, who handled education sessions? Who did health and safety assessments?
    **Response:**

We acknowledge and appreciate this comment. COVID-19 mitigation activities were done by the US Mission India as a team involving multiple agencies/divisions within the US Embassy as mentioned in section “COVID-19 mitigation activities undertaken at US Mission in India”. Case investigation and contact tracing program were collaboration between Health Unit and US CDC.

18. Vaccination – Who decided what vaccine to be given to whom? Please mention that information.
    **Response:**

We acknowledge and appreciate this comment. Since this is a secondary data analysis, so, we are analyzing the pre-collected data. As clarified in limitations, some variables of interest were not available. We did not mention this as this was not focused part of our analysis.

19. Case investigation and contact tracing – what is government-recommended isolation or quarantine. There are four state governments and one federal government involved. Explain which guideline and give references.
    **Response:**

We acknowledge and appreciate this comment. We have incorporated the requested changes. Please note that as we have clarified that the guidance picked for our patients was strictest out of US CDC, Government of India (MOHFW) and Indian State Governments to comply with the laws. Plus, this kept on changing from time to time with different variants of COVID-19 and published guidelines changes.

20. Difference in AR – p-value is given only in the text. It would be better to give in the table as well.
    **Response:**

We acknowledge and appreciate this comment. The p-values are kept in the text in order to avoid making the table more congested. It also helped to summarize the overall difference in the result section.

21. When there is overall attack rate, why AR by age and gender is not given? Is there a reason? If yes, please mention it.

**Response:**

We acknowledge and appreciate this comment. Since this is a secondary data analysis, so, we are analyzing the pre-collected data. As clarified in limitations, some variables of interest were not available. So, we could not calculate the age and gender wise ARs.

Thank you once again for your valuable comments. We believe that the revisions have strengthened our manuscript, and we look forward to your favorable consideration for publication.

Sincerely,

Dr Jaspreet Singh

US Embassy

New Delhi
